# Total Joint Replacement for Immediate Reconstruction following Ablative Surgery for Primary Tumors of the Temporo-Mandibular Joint

**DOI:** 10.3390/jpm13071021

**Published:** 2023-06-21

**Authors:** Luis-Miguel Gonzalez-Perez, Jose-Francisco Montes-Carmona, Eusebio Torres-Carranza, Pedro Infante-Cossio

**Affiliations:** 1 Department of Oral and Maxillofacial Surgery, Virgen del Rocio University Hospital, 41013 Seville, Spain; josmoncar@gmail.com (J.-F.M.-C.); drtorres@clinicatorrescarranza.es (E.T.-C.); pinfante@us.es (P.I.-C.); 2 Department of Surgery, School of Medicine, University of Seville, 41009 Seville, Spain

**Keywords:** temporomandibular joint, primary tumors, diagnosis, surgical treatment, temporo-mandibular joint replacement, joint prosthesis

## Abstract

Temporomandibular joint (TMJ) tumors are rare and difficult to diagnose. The purpose of this retrospective study was to evaluate the clinicopathologic characteristics of twenty-one patients with primary TMJ tumors between 2010 and 2019 and to analyze the surgical outcome and morbidity after ablative surgery and TMJ replacement. This case series confirmed the difficulty of diagnosis and reaffirmed the need for early recognition and management of TMJ tumors. There were no pathognomonic findings associated with TMJ tumors, although single or multiple radiopaque or radiolucent areas were observed on plain or panoramic radiographs. Occasionally, bone resorption or mottled densities caused by pathologic calcification and ossification were seen. Computed tomography and magnetic resonance imaging played an important role in the diagnosis. In our study, the distribution of histologic types of TMJ tumors was quite different from that of other joint tumors. The recommended treatment was surgical intervention by ablation of the joint and TMJ replacement. The results of this retrospective study support the surgical exeresis and replacement with TMJ stock and custom-made prostheses and show that the approach is efficacious and safe, reduces pain and improves mandibular movements, with few complications.

## 1. Introduction

Primary tumors of the temporomandibular joint (TMJ) are not very common and often mimic common conditions, such as masticatory myalgia or internal derangement, leading to a delay in diagnosis and, therefore, to a delayed therapeutic option [1,2,3,4,5,6,7,8]. Radiographically, no pathognomonic findings are associated with TMJ tumors, although single or multiple radiolucent or radiopaque areas can be seen on plain or panoramic studies. Evidence of bone destruction is often present, and mottled densities caused by pathological calcification and ossification can occasionally be seen. Computed tomography (CT) and magnetic resonance imaging (MRI) play an important role in the diagnosis of these entities. The distribution of histological types of primary TMJ tumors is quite different from other articular tumors, with a predominance of benign histological forms. Their clinical presentation in the mandibular condyle usually consists of a combination of preauricular pain and impaired temporo-mandibular function with mandibular movement limitation, dentofacial deformity and occlusal asymmetry [9,10,11,12,13,14,15]. Resection and replacement of the TMJ is usually reserved for patients with irreversible damage, as in tumoral pathology [16,17,18,19,20].

The objective of this retrospective study was to investigate the clinical, radiological and histopathological characteristics of patients with primary TMJ tumors who were managed with ablative surgery and immediate reconstruction with total joint replacement and to evaluate their surgical outcome and morbidity.

## 2. Materials and Methods

Twenty-three patients with TMJ tumoral pathology were referred to the Department of Oral and Maxillofacial Surgery of the Virgen del Rocio University Hospital of Seville, Spain, between January 2010 and January 2019. The cases studied were adult male and female patients referred to the outpatient clinic with a primary TMJ tumor who were treated with ablative surgery and immediate reconstruction with total joint replacement. The following inclusion diagnostic criteria were assessed: (1) a history of persistent and significant pain and functional impairment; (2) a clinically and radiographically documented tumoral history. The exclusion criteria were: (1) patients without 5 years of follow-up, (2) infective disease at the implantation site, (3) insufficient quantity of bone support; (4) documented allergy of any of the prosthetic materials. Two patients, one with metastatic carcinoma and one with extraarticular location, were excluded from the study.

Twenty-one patients who met the inclusion criteria, nine males (43%) and twelve females (57%), were included in the study. All patients were initially investigated with plain radiographs. CT and/or MRI scans were performed in all the cases included in this study. Bone scintigraphy was performed in 4 cases. The mean preoperative period from initial TMJ symptoms to surgical treatment was 1 year (range: 6 months to 4 years).

The recommended treatment was surgical intervention by ablation of the joint and TMJ replacement. In all cases, surgical procedure was performed under general anesthesia with nasotracheal intubation. After the preauricular approach and the Al-Kayat–Bramley incision, a condylectomy was performed for the removal of the tumor. The glenoid fossa was then flattened, and the fossa was adapted and inserted. All surgeries were performed using the Zimmer Biomet Microfixation TMJ Replacement System^®^, Jacksonville, FL, USA (stock and custom-made prosthetic systems), and all procedures replaced the glenoid fossa component and the mandibular condyle. The fossa and mandibular components were available in three different sizes in the stock prosthetic system. The mandibular component of the prosthesis was manufactured from a cobalt–chromium–molybdenum (Co-Cr-Mb) alloy with a roughened titanium plasma coating on the host bone side of the ramal plate for increased bony integration. The Co-Cr-Mb alloy was type 99 of the American Society for Testing and Materials (ASTM). The fossa prosthesis was made of ultra-high-molecular-weight polyethylene (UHMWPE). In the stock TMJ replacement, templates were used intraoperatively to determine the fit, and then, the final TMJ prosthesis was inserted. The precision of a custom-made prosthesis makes the use of TMJ templates unnecessary. The screws used in the procedure were made of 6Al/4V titanium alloy. Intermaxillary fixation was temporary performed to restore the vertical dimension and dental occlusion. When the desired position was reached, the templates were replaced for the final prosthetic components.

The change in pain intensity (preoperative vs. current) was evaluated using a visual analog scale (VAS, 1 to 10), with higher scores indicating more severe pain. Jaw opening was measured in centimeters with a Therabite rule between incisal edges of maxillary central incisors. The signs assessed as indicators of the efficacy of surgical treatment were a significant reduction in pain at rest of 4 points or more, an improvement in temporomandibular function, and recovery of normal ranges of mandibular opening.

Active opening jaw motion initiated by patient’s masticatory musculature was started immediately postoperatively. A soft food diet was recommended for the first month and normal sustenance thereafter. At follow-up, all patients were asked about any limitation in the activities of daily living and were examined for range of motion of TMJ or any neurovascular deficit. Imaging studies were carried out immediately after the operation and at 5-year follow-up for evaluation. All patients with TMJ tumors underwent radiological studies every 3 months during the first 2 years and every 6 months beginning the third postoperative year, and a CT scan was obtained in those with persistent symptoms or suspected recurrent TMJ lesions. Surgical morbidity and prosthetic implant survival were documented.

## 3. Results

**Figure 1:**
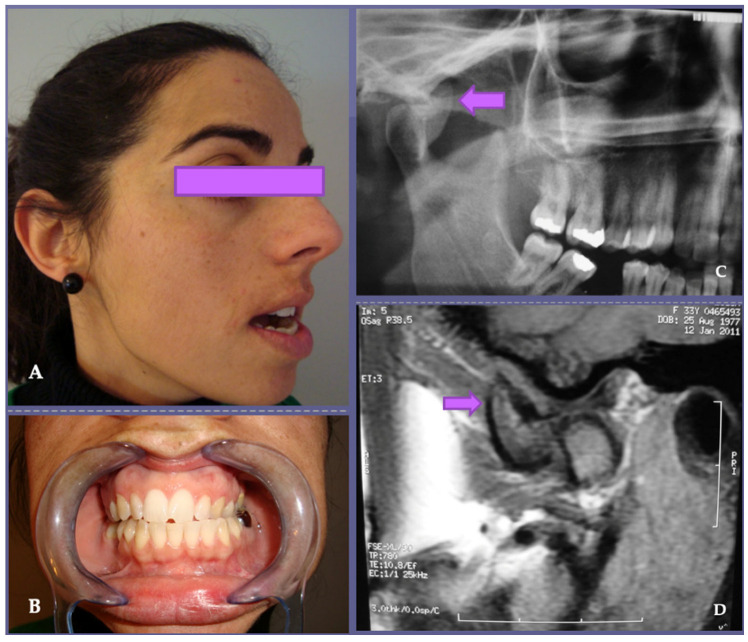
Osteochondroma is one of the most common benign tumors of the axial skeleton but is rarely found in the TMJ. (**A**) Its clinical presentation in the mandibular condyle, as in our 5th case, usually occurs with a combination of preauricular pain, mandibular dysfunction and facial asymmetry. When considering surgical risks involved in tumoral exeresis and temporomandibular reconstruction, the differential diagnosis is of great importance. (**B**) In this patient, an osteochondroma was suspected given the clinical characteristics of the lesion; restricted mouth opening and changes in occlusion with unilateral posterior open bite and contralateral crossbite were related to the anatomical location of the tumor and were due to alterations in the vertical dimension. Orthopantomography (**C**) and magnetic resonance imaging (**D**) showed an osteochondroma with growth arising from the anterolateral aspect of the right mandibular condyle (see arrows), distinguishing it from condylar hyperplasia, seen as an enlargement of the condylar process.

**Figure 2:**
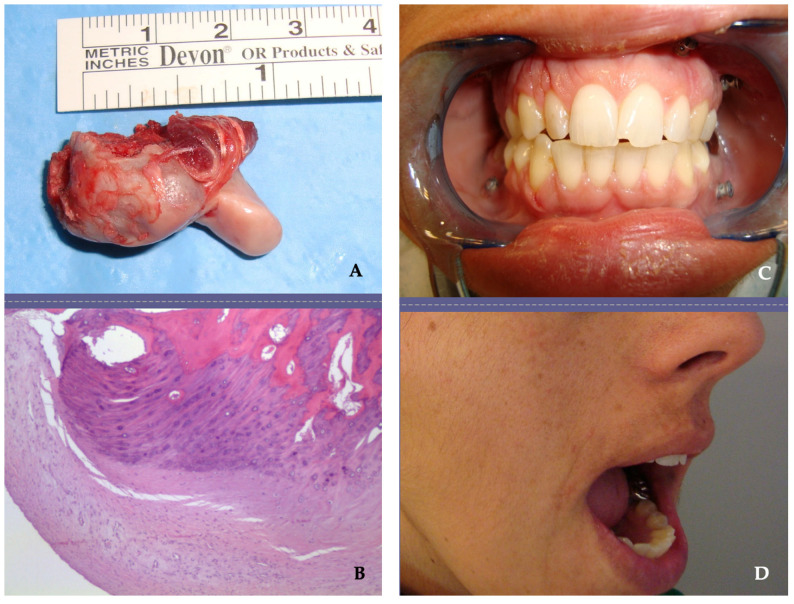
(**A**) Surgical resection of clinical case shown in Figure 1. The tumor showed no infiltration of the surrounding tissues. (**B**) Histopathological diagnosis was osteochondroma. (**C**,**D**) Postoperative view of the patient shows correction of the occlusal alterations and increased mouth opening after surgery.

**Figure 3:**
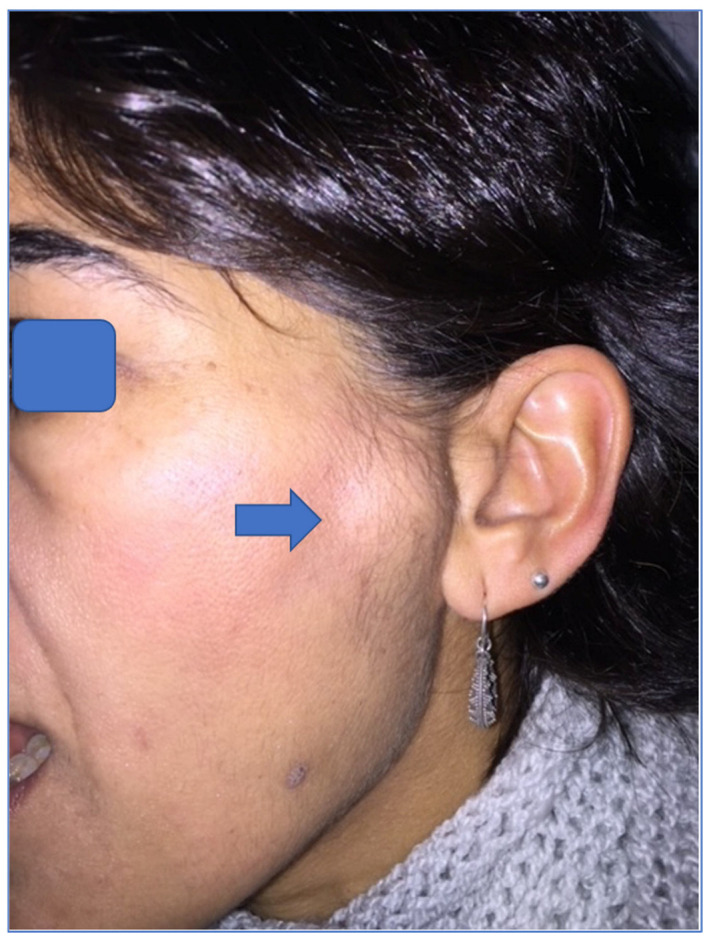
Painful and slow-growing TMJ osteochondroma in the left preauricular area (see arrow), with facial asymmetry, accompanied by ipsilateral open bite and right mandibular lateral deviation in our patient (case number 17).

**Figure 4:**
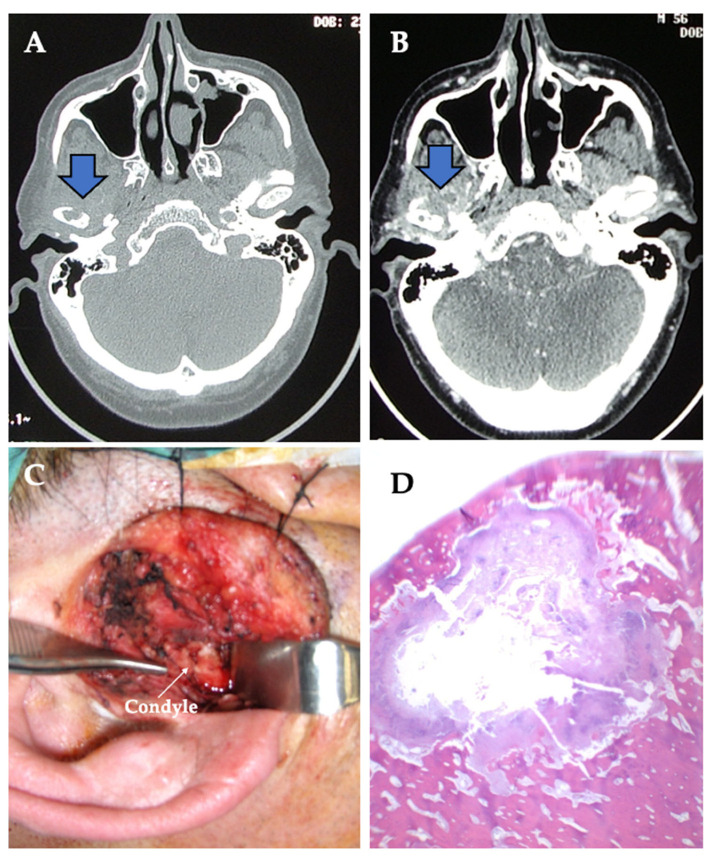
(**A**,**B**) Computed tomography, without and with contrast, shows well-delimited lytic lesion involving right mandibular condyle (see arrows), and is recommended as the imaging study of choice when planning a surgical treatment. (**C**) The TMJ tumor appeared well encapsulated with an expansile intramedullary lesion (see arrow). The surgical TMJ defect was replaced with a total prosthesis. (**D**) The tumor in the condylar specimen was whitish and hard elastic with a central cavitation. The margins of surgical resection appeared without tumor. There was no extraosseous infiltration. The histopathological diagnosis was of primary intramedullary chondrosarcoma (case number 3).

The clinical, radiological and histopathological characteristics of the 21 patients are summarized in Table 1. The mean age was 54 years (range: 29–72 years). The main reasons for the consultation were mandibular deviation in 8 cases and posterior open bite in 7 cases (Figure 1, Figure 2, Figure 3 and Figure 4). Less frequent were asymmetric prognathism (2 cases), preauricular swelling (2 cases) and TMJ dysfunction (2 cases). Radiologically, 13 cases presented as radiopaque lesions, 3 cases showed radiopaque areas, 2 cases radiolucent areas and 2 cases mottled densities. One case of bone destruction due to a malignant lesion (chondrosarcoma) was found.

After TMJ replacement, all the patients were followed up for at least 5 years (range: 5–10 years). Mean pain (VAS) and preoperative opening (cm) were 5.9 (range: 4–8) and 3.5 (range: 2.4–4.8), respectively. Mean pain (VAS) and postoperative opening (cm) measured at 5 years were 1 (range: 0–4) and 4.2 (range: 3–5.3), respectively. Therefore, a pain reduction of 4.9 points on the VAS scale and a postoperatively increased mouth opening of 0.7 cm were observed. Pain intensity was reduced by 83%. Jaw opening was improved by 20%.

**Figure 5:**
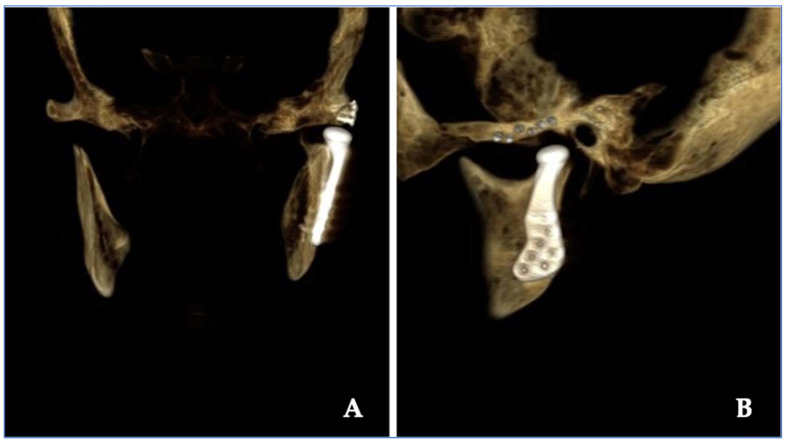
Postoperative tridimensional computed tomography reconstruction of clinical case shown in Figure 3. (**A**) Coronal view. (**B**) Sagittal view.

**Figure 6:**
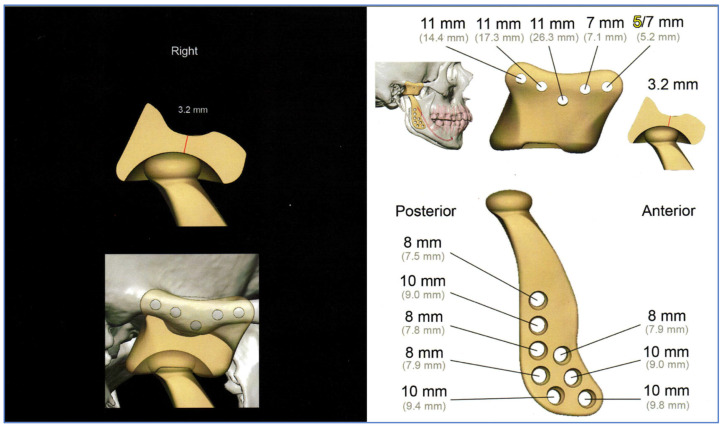
Custom-made prosthesis, designed for one of our cases, using the patient’s DICOM data.

Resection margins were wide in all cases (Figure 2 and Figure 4). All diagnoses were confirmed by anatomopathological study. Most of the histopathological diagnoses were osteochondromas in 15 cases (71% of our studied population). Three cases were osteomas and one each of chondroblastoma, chondromyxoid fibroma and chondrosarcoma. A total of 24 joints (18 unilateral and 3 bilateral) were operated on, and consequently, 24 TMJ prostheses were fitted. All surgeries were performed with the Zimmer Biomet Microfixation TMJ Replacement System^®^, Jacksonville, FL, USA, replacing both the skull base component (glenoid fossa) and the mandibular condyle. Twenty-two stock prostheses and two custom-made prostheses were implanted (Figure 5 and Figure 6). Occlusal equilibration was required in one patient with persistent premature occlusal contacts. No particular predilection for tumor location within the TMJ was observed (left TMJ: 13 cases, right TMJ: 11 cases).

Comparing stock and customized groups, no statistically significant differences were detected with respect to reduction in pain intensity and improvement in maximum mouth opening compared, although these data should be considered carefully given the small number of cases in the customized prostheses group.

Functional and oncological results of the surgery were good. No patient reported 7th nerve dysfunction after 3 months. Two of twenty-four implants (TMJ prostheses) were explanted during the study period of 10 years, as a result of instability of the implant for screw loosening and metal hypersensitivity. The patient’s satisfaction with the clinical outcome was 9 on a scale of 1 to 10. Recurrence has not been reported in our patients.

## 4. Discussion

The temporomandibular joint (TMJ) is one of the most complex and widely used joints and is the only paired joint of the skeletal system. The most frequent pathologies of the TMJ are dysfunctional disorders, internal derangement, masticatory myalgias and degenerative arthropathies, which cause considerable structural and functional abnormalities leading to early diagnosis. Primary tumors of the TMJ are rare and represent a diagnostic enigma due to their non-specific clinical course and radiographic presentation. There is little literature available on their characteristics and outcomes; thus, experience in the treatment of tumors and tumoral lesions in this anatomical area is limited. There are several types of tumors that can affect the TMJ, as any other joint, including benign tumors, such as osteochondroma (Figure 1, Figure 2 and Figure 3), osteoma, osteoblastoma, chondroma, chondroblastoma, non-ossifying fibroma, hemangioma or lipoma, as well as malignant tumors such as synovial sarcoma, osteosarcoma, Ewing sarcoma or chondrosarcoma (Figure 4) [1,2,5,9,11,21,22,23,24,25,26,27,28,29,30,31]. The wide variety of primary tumors of the TMJ indicates the great range of possible treatments, with most of them based on surgical intervention (Table 2).

The classification of TMJ tumors plays a key role in the understanding, diagnosis, and treatment of these rare clinical conditions. Since these tumors can present with a wide variety of histological and clinical characteristics, their classification is essential to establish a multidisciplinary and collaborative approach in clinical practice between maxillofacial surgeons, pathologists, radiologists, and oncologists, and it is essential to ensure optimal management of these complex cases (Table 2).

In our study, osteochondroma was the most common neoplasm affecting the TMJ [3,4,7,11,12,13,14,15]. Osteochondroma is one of the most common benign tumors of the axial skeleton but is rarely found in the TMJ. Its clinical presentation is in the mandibular condyle, as observed in our case series. Primary TMJ tumors are rare lesions with a histopathological profile quite different from that seen in other articular areas, and they often mimic common conditions of the TMJ, such as TMJ dysfunctional syndrome and neurologic or otologic pathologies, leading to a delay in diagnosis and surgical treatment. For this reason, a high index of suspicion is needed for the timely diagnosis and management of tumors at this rare site. This may lead to earlier intervention and may improve outcome [3,4,5,7,8,9,10,12,13,14,15,21,23,27]. In some patients, benign tumors of the TMJ remain as totally asymptomatic lesions. In patients who have intense pain that does not respond to conservative treatment for more than one year, radiographic imaging may be considered to rule out these tumors. Changes in occlusion with ipsilateral posterior open bite and contralateral posterior crossbite are related to the anatomical location of the tumors and are due to alterations in the vertical dimension (Figure 1 and Figure 3). These occlusal disorders disappeared after surgical treatment (Figure 2). Considering the surgical risks involved in tumor removal and temporomandibular reconstruction, prior differential diagnosis was of great importance. In our cases, osteochondroma was suspected given the clinical characteristics of the lesion. The changes in occlusion with unilateral posterior open bite and contralateral crossbite were related to the anatomical location of the tumor and were due to the alteration of the vertical dimension [4,5,6,10,11,12,13,14,15,16,17,18,19,20,21,22,23,24,25,26,27].

Surgical treatment of TMJ tumors depends on several factors such as the type, size, location, and extent of the tumor, as well as the patient’s overall health and individual circumstances. In general, surgical treatment involves the removal of the tumor as well as any affected surrounding tissue. The goal of surgery is to completely remove the tumoral lesion while minimizing damage to the surrounding structures of the mandible and other facial structures. Treatment of TMJ tumors should mostly include partial or complete resection, the oncological and functional results of which are good [2,3,4,5,6,7,10,11,14,15,31]. In our series, which had a relatively large number of cases and a minimum follow-up of 5 years, there were no recurrences. Immediate reconstruction after resection of a primary TMJ tumor is a good strategy to avoid a second operation.

The visual analogue scale (VAS) on pain was measured at rest in our analysis. TMJ pain in end-stage TMJ disease is often a dull, constant and severe long-standing ache that is aggravated, in a very variable way according to the patients, by mandibular opening or mastication. There is usually a complaint of reduced jaw mobility preoperatively and frequent grinding noise within the TMJ associated with mandibular movement or chewing. The reason for our results in jaw opening is that although none of the surgical procedures were carried out in patients who had good mandibular motion, six patients included in the tumoral group (28% of 21 patients) had a preoperative normal mouth opening ≥ 4 cm, and although strictly speaking, there was no limited mouth opening, all of them were cases with a history of progressive mandibular deviation, open bite on the ipsilateral side and posterior crossbite on the contralateral side.

In our study, primary malignant tumors of the TMJ were unusual. They can invade sensory and motor nerves, causing paresthesia and paralysis in the TMJ area, mandible and lower lip. The eighth cranial nerve may be involved; thus, changes in hearing, tinnitus, or dizziness should be monitored. Changes in mandibular function are common, as in case number 3 in our study (Figure 4); thus, any abrupt change in occlusion, trismus, and pathologic fractures should be considered as a sign of malignancy. Pain is not a symptom indicative of malignant neoplasm, but it may occur in this TMJ area as an extension of other tumors, easily confused with myofascial pain syndrome and TMJ internal disorders, which in many cases have hindered early diagnosis of malignant disease. The most common, but outside the scope of our study, are metastatic tumors of the breast, lung, thyroid, prostate, stomach, skin, ovaries, colon and kidney, although maxillofacial, nasopharyngeal, and intracranial tumors that may metastasize to the TMJ should also be considered [5,9].

Primary intrinsic malignancies of the TMJ include chondrosarcoma (only one out of all our cases), osteosarcoma, osteochondrosarcoma, synovial sarcoma, fibrosarcoma and epidermoid carcinoma. Other tumors described with condylar involvement include multiple myeloma, solitary plasmacytoma or reticulosarcoma (bone malignant lymphoma). Chondrosarcoma, which usually occurs primarily in the long bones, presents as a rapidly growing asymptomatic form in the preauricular area. Pain, when it occurs, is caused by compression of the adjacent anatomical structures [5,9].

The management of TMJ reconstruction after ablative tumor surgery remains a challenge. In some cases, reconstructive surgery may be necessary to repair any damage to the mandible or craniofacial structures caused by the tumor or by surgery. This may involve the use of bone grafts or other materials to restore the temporo-mandibular structure and function. Surgery is often considered as an option of last resort. However, there are instances where surgery is the definitive and sometimes only treatment option. TMJ specialists must be prepared to recognize and manage disorders that present with more complex cases where TMJ surgery is less clear. While the diagnosis and surgical aims of severe tumoral cases are straight forward, when it comes to initial cases, the diagnosis is often complicated, and the surgical perspective is less clearly defined, especially when TMJ disease progression is slow. However, it can ultimately lead to end-stage TMJ disease with, as a consequence, significant disability requiring surgical intervention, as occurs in patients of this study [3,7,8,10,12,13,15,16,17,19,20,21,22,23,28,29,30].

To the best of our knowledge, the main surgical indication for a total TMJ prosthesis is the presence of a symptomatic severely damaged TMJ with extensive joint destruction (when nothing is salvageable), which can result from either different types of severe TMJ tumor disease or failed previous surgeries, and therefore, it is wrong not to consider surgical definitive treatment modality of collapsed articular cartilage and degenerative changes that severely interfere with the smooth, painless movement of the TMJ. When compared to other surgical reconstructive procedures, such as costochondral or sternoclavicular grafts, the use of TMJ prosthesis can reduce the duration of surgery and hospitalization time, which provides immediate function without intermaxillary fixation postoperatively and no morbidity from a donor site. The prosthetic replacement may also present some disadvantages such as loss of protrusion and laterality movements due to the detachment of the lateral pterygoid muscle during surgery, fracture of the TMJ prosthesis from metal fatigue, loosening of screws, etc. [12,15,20].

The surgical placement of TMJ prostheses, as it happened in our cases, provides significant reduction in pain intensity and secondary TMJ dysfunction to tumoral pathology of the TMJ (Figure 5 and Figure 6). The appropriate selection of the case is the most important requirement for a successful surgical intervention to achieve the desired outcomes of relief of symptoms and improved function [3,4,7,10,11,12,13,14,15,17,19]. In our case series, the functional and oncological results of surgery were good.

The quantification of implant failures is a determining factor with prognostic value, enabling the clinician to objectively quantify the success of the surgical treatment [3,7,8,10,12,13,15,16,17,19,20,21,22,23]. Although there were complications necessitating the removal of the prosthesis (2 out of 24 prostheses: 8% of our studied population), there were no device-related mechanical failures; and one case of TMJ prosthesis was explanted due to malocclusion, which was a result of loosening of the implant screws. Another case was explanted due to severe hypersensitivity to the metal alloy not detected before surgical replacement, with only the mandibular prosthetic component replaced. Many factors contribute to the success or failure of a total TMJ replacement. These factors include prosthesis micromovements, loosening of prosthetic components, allergic reaction and metal hypersensitivity, material wear breakdown and corrosion, bacterial contamination, and the development of heterotopic bone apposition around the prosthesis [3,5,8,10,12,22,28]. In our series, only two prostheses had to be removed, even though the result was satisfactory for patients.

Two types of prosthetic implants were used in our study: stock TMJ replacement in which the surgeon must fit it to the implantation area, as occurred in 19 of our patients, and custom-made prostheses, which are made specifically for each clinical case, as had occurred in two of our patients.

The stock TMJ replacement must be compatible with a range of different patient geometries and anatomies, and typically a range of different sizes is necessary, such as large (55 mm), medium (50 mm) and small (45 mm) condyle–ramus components. Mainly, implant failure was a consequence of wear. The biomaterials from which prosthetic implants are made must be biocompatible, and any wear particles produced must be compatible with the body and must not cause adverse biological reactions, but the wear of materials is unpredictable. Similarly, the bone quality of patients varies considerably, and the methods of fixation must be able to accommodate different bone interface conditions [10,14,15,17,19,20,22].

One problem in planning surgery is the inability to predictably create complex prosthetic contours using commercially available stock prostheses. These devices are supplied as generic sizes and shapes designed on the basis of the average patient. In more complex and severe clinical cases, the surgeon may need prolonged operative time shaping the prosthesis to customize the stock prosthesis to fit the patient’s bone contours, and these repeated manipulations to adapt the prosthesis to difficult anatomical conditions may cause prosthetic fracture due to material fatigue. One solution to this deficiency is to use computer-guided surgical planning technologies to create a passive fitting replacement designed for specific anatomical requirements. To date, the most common application of additive manufacturing has been the fabrication of patient-specific models (Figure 6), which are created for pre-operative planning using patient-specific imaging data in DICOM files (Digital Imaging/Communications in Medicine), which are then converted into stereolithography files, the standard manufacturing format used to print patient-specific cranio-facial models. The use of these medical models enables planning and simulation of surgery and manually pre-shapes commercially available prostheses. Recent advances in additive manufacturing allow for the prefabrication of patient-specific customized prostheses using the patient’s DICOM data. The advantages of rapid prototyping in designing and manufacturing customized prostheses are that they do not require intraoperative modifications, and they provide a better passive fitting.

Considering the implanted material, which may invoke a tissue response, the materials used remain basically the same as before, namely, metal alloys and polymers (mostly polyethylene). Bioactive coatings and particulate materials constitute the last broad category to which the body reacts in joint replacement. Employing the most advantageous characteristics of biocompatible materials is an essential consideration in the design and manufacturing of any replacement device. In our cases, cobalt chromium alloy, with its undersurface coated with a roughened titanium plasma spray for increased osteointegration, contributes to its strength and biocompatibility. Its excellent wear characteristics when articulated against a UHMWPE material presently make it the standard for the non-moveable articulating surface of most orthopedic total joint replacement devices [12,15,17,19,20] (Figure 5 and Figure 6).

The results of this case series support the need for further research breakdown of this form of surgical treatment in a rigorously controlled prospective analysis. Despite the fact that this case series includes a relatively high number of cases, the results should be taken with caution, and further long-term follow-up studies with clear results are still needed.

## 5. Conclusions

This was a retrospective study in a single center. The relatively large number of patients operated on and the standard operative and perioperative management made our study consistent and facilitated comparison with other studies. Temporomandibular joint (TMJ) tumors are rare lesions, with a histopathological profile quite different from that seen in other maxillofacial areas, and they often mimic common TMJ conditions, resulting in delayed diagnosis. There is often a long delay between the onset of initial symptoms and definitive diagnosis. A high index of suspicion is needed for timely diagnosis and treatment of tumors in these rare areas by integrating the components of patient history, clinical presentation, and imaging findings. This may lead to earlier intervention and a better outcome. Osteochondroma is the most common tumor in the TMJ. Changes in occlusion with ipsilateral posterior open bite and contralateral posterior crossbite are related to the anatomic location of the tumors and are due to alterations in the vertical dimension. These occlusal disorders disappeared after surgical treatment. Treatment of TMJ tumors should mostly include partial or complete resection, the oncological and functional results of which are good. The surgical placement of TMJ prostheses provides a significant reduction in pain intensity and TMJ dysfunction secondary to tumoral pathology of the TMJ. In our study, the main indication was a damaged TMJ with extensive destruction, which was the consequence of different tumors. When compared to other surgical reconstructive procedures, such as costochondral or sternoclavicular grafts, the use of a TMJ prosthesis can reduce the duration of surgery and hospitalization time and provides immediate function and no morbidity from a donor site. TMJ replacement is often considered to be the last resort in the surgical treatment of TMJ disorders and can be recognized as the gold standard treatment in immediate reconstruction after ablative surgery for primary tumors of TMJ.

## Figures and Tables

**Table 1:** Clinical, radiological and histopathological characteristics of patients.

Nº	Age/ Sex	Clinical Signs	Imaging Characteristics	Preop Pain *	5 yr Postop Pain *	Preop Opening **	5 yr Postop Opening **	Histological Diagnosis	Prosthesis Side
1	62/F	Mandibular deviation	Radiopaque lesion	4	0	2.5	3.5	Osteochondroma	Right
2	57//F	Posterior open bite	Radiopaque lesion	5	0	3	3	Osteochondroma	Left
3	57/M	Posterior open bite	Bone destruction	8	0	4.2	4.8	Chondrosarcoma	Right
4	60/F	Mandibular deviation	Radiopaque lesion	5	0	3.2	3.8	Osteochondroma	Left
5	49/M	Preauricular swelling	Radiopaque area	6	0	3.8	4.1	Osteochondroma	Right
6	67/F	Mandibular deviation	Radiopaque area	5	4	4	4	Osteoma	Right
7	65/F	Posterior open bite	Radiopaque area	7	2	3.5	3.7	Osteochondroma	Left
8	59/F	Asymmetric prognathism	Radiopaque lesion	4	0	4.2	4.5	Osteoma	Left
9	51/F	Posterior open bite	Radiopaque lesion	4.5	0	2.4	3.6	Osteochondroma	Right
10	59/F	Mandibular deviation	Radiopaque lesion	5	2	3.4	4.5	Osteoma	Left
11	52/M	TMJ dysfunction	Radiolucent areas	6	1	3.7	4.8	Osteochondroma	Bilateral
12	53/F	Preauricular swelling	Radiopaque lesion	6	2	3.1	3.6	Chondroblastoma	Left
13	68/M	Mandibular deviation	Radiolucent area	8	2	3.7	4.4	Osteochondroma	Left
14	30/F	TMJ dysfunction	Mottled densities	6	2	4.3	5	Osteochondroma	Right
15	48/M	Mandibular deviation	Mottled densities	5	0	4.8	5	Chondromyxoid fibroma	Right
16	55/F	Asymmetric prognathism	Radiopaque lesion	5	0	2.9	3.6	Osteochondroma	Left
17	38/M	Mandibular deviation	Radiopaque lesion	6	0	4.6	5.3	Osteochondroma	Left
18	56/M	Posterior cross-bite	Radiopaque lesion	6	1	4.1	4.1	Osteochondroma	Right
19	43/M	Posterior cross-bite	Radiopaque lesion	6	0	4	4.5	Osteochondroma	Bilateral
20	29/F	Posterior cross-bite	Radiopaque lesion	7	1	3.1	4.4	Osteochondroma	Bilateral
21	72/M	Mandibular deviation	Radiopaque lesion	7	3	2.7	3.5	Osteochondroma	Left

Abbreviations: M, male; F, female; Yr, years. * Pain intensity measured by VAS, Visual Analogue Scale. ** Mandibular opening measured in centimeters (cm). Preop, preoperative; Postop, postoperative.

**Table 2:** Primary tumors of the temporo-mandibular joint classification.

	Benign	Aggressive	Malignant
Bone formers	Osteoid osteoma	Osteoblastoma	Osteosarcoma
	Osteoma		
Cartilage formers	Osteochondroma	Chondroblastoma	Chondrosarcoma
	Chondroma (enchondroma and periosteal chondroma)		Osteochondrosarcoma Synovial sarcoma
Fibrous tissue formers	Chondromyxoid fibroma	Desmoid (aggressive fibromatosis)	Malignant fibrous histiocytoma
			Fibrosarcoma
Round cell			Ewing’s sarcoma
			Primitive neuroectodermal tumor
Myelogenous		Eosinophilic granuloma (histiocytosis X, Langerhans cells)	Myeloma Solitary plasmacytoma
			Reticulosarcoma (bone malignant lymphoma)
Lipogenic	Lipoma		Liposarcoma
Myogenic			Leiomyosarcoma
			Rhabdomyosarcoma
Vascular	Hemangioma		Angiosarcoma
Neurogenic	Neurilemmoma		
Unfilial lineage		Giant cell tumor	Chordoma
			Adamantinoma
Pseudotumoral lesions	Non-ossifying fibroma	Osteomyelitis	
	Cortical fibrous defect	Paget’s disease	
	Essential bone cyst	Pigmented villonodular synovitis	
	Aneurysmal bone cyst	Synovial chondromatosis	
	Fibrous dysplasia		
	Bone infarction		
	Myositis ossificans		
	Brown tumor of hyperparathyroidism		

## Data Availability

The data presented in this study are available by contacting the corresponding author upon reasonable request.
